# Supplementary material for: ProteinShader: illustrative rendering of macromolecules
Source: BMC Struct Biol. 2009 Mar 30;9:19. doi: 10.1186/1472-6807-9-19 (PMC2672931; doi:10.1186/1472-6807-9-19)
Supplement: Additional file 1 — ProteinShader program without source code. This compressed file contains the complete ProteinShader program including associated libraries, but no source code. A README.txt file gives an overview of the ProteinShader distribution, and the index.html file in the help subdirectory has directions on getting started with the program as well as a set of tutorials. [file 1472-6807-9-19-S1.zip › ProteinShader-beta-0_9_4-binary/help/api/org/proteinshader/structure/visitor/class-use/Visitor.html]

Uses of Class org.proteinshader.structure.visitor.Visitor (ProteinShader API)


|  |  |  |
| --- | --- | --- |
| **Overview** \| **Package** \| **Class** \| **Use** \| **Tree** \| **Deprecated** \| **Index** \| **Help** | |  |
| PREV   NEXT | **FRAMES**    **NO FRAMES**     **All Classes** |  |


---


## **Uses of Class org.proteinshader.structure.visitor.Visitor**

| Packages that use Visitor | |
| --- | --- |
| **org.proteinshader.structure** | Holds the classes that store information from a Protein Data Bank file: Structure, Model, Chain, AminoAcid, Heterogen, Water, Atom, Bond, Helix, BetaStrand, Loop, *etc*. |
| **org.proteinshader.structure.visitor** | Holds Visitor classes that know how to traverse the hierarchy of objects held by class Structure. |

Uses of Visitor in org.proteinshader.structure

| Methods in org.proteinshader.structure with parameters of type Visitor | |
| --- | --- |
| `void` | `AminoAcid.accept(Visitor visitor)`             Accepts a Visitor and does a callback. |
| `void` | `Water.accept(Visitor visitor)`             Accepts a Visitor and does a callback. |
| `abstract  void` | `Drawable.accept(Visitor visitor)`             Accepts a Visitor and does a callback. |
| `void` | `Segment.accept(Visitor visitor)`             Accepts a Visitor and does a callback. |
| `void` | `Structure.accept(Visitor visitor)`             Accepts a Visitor and does a callback. |
| `abstract  void` | `Residue.accept(Visitor visitor)`             Accepts a Visitor and does a callback. |
| `void` | `Chain.accept(Visitor visitor)`             Accepts a Visitor and does a callback. |
| `void` | `Model.accept(Visitor visitor)`             Accepts a Visitor and does a callback. |
| `void` | `Helix.accept(Visitor visitor)`             Accepts a Visitor and does a callback. |
| `void` | `Atom.accept(Visitor visitor)`             Accepts a Visitor and does a callback. |
| `abstract  void` | `Region.accept(Visitor visitor)`             Accepts a Visitor and does a callback. |
| `void` | `BetaStrand.accept(Visitor visitor)`             Accepts a Visitor and does a callback. |
| `void` | `Loop.accept(Visitor visitor)`             Accepts a Visitor and does a callback. |
| `void` | `Bond.accept(Visitor visitor)`             Accepts a Visitor and does a callback. |
| `void` | `Heterogen.accept(Visitor visitor)`             Accepts a Visitor and does a callback. |

Uses of Visitor in org.proteinshader.structure.visitor

| Subclasses of Visitor in org.proteinshader.structure.visitor | |
| --- | --- |
| `class` | `AminoAcidLabelVisitor`             Traverses the AminoAcids of a Structure and builds a non-redundant list of residueIDs. |
| `class` | `BondDestroyerVisitor`             Removes Bonds on all Atoms that it visits. |
| `class` | `BondGeneratorVisitor`             Generates standard Bonds for AminoAcids and Waters. |
| `class` | `BoundsVisitor`             Knows how to find the min and max xyz-coordinate values for all Atoms in each Model. |
| `class` | `FrenetFrameGeneratorVisitor`             Calculates a discrete Frenet frame for each AminoAcid visited. |
| `class` | `LoopGeneratorVisitor`             Generates Loop objects from any AminoAcids of a Chain that do not already belong to a Helix or BetaStrand. |
| `class` | `ModifierVisitor`             Knows how to modify multiple Drawable objects while traversing the hierarchy of objects contained in a Structure. |
| `class` | `SFWriterVisitor`             A simple file writer subclass of Visitor that writes to a file all of the data from each Model in a Structure. |
| `class` | `VisibilityVisitor`             Knows how to traverse the Structure object hierarchy and find all visible Drawable objects. |

| Methods in org.proteinshader.structure.visitor with parameters of type Visitor | |
| --- | --- |
| `void` | `Visitable.accept(Visitor visitor)`             Accepts a Vistor so that a call back can be done. |

---


|  |  |  |
| --- | --- | --- |
| **Overview** \| **Package** \| **Class** \| **Use** \| **Tree** \| **Deprecated** \| **Index** \| **Help** | |  |
| PREV   NEXT | **FRAMES**    **NO FRAMES**     **All Classes** |  |


---

# *Copyright © 2007-2008*
